# A Stiff Finger: A “Concrete” Diagnosis

**DOI:** 10.7759/cureus.20425

**Published:** 2021-12-15

**Authors:** Laura Bateman, Iain Roy, Olivia Kenyon, Robert Manton, Patrick Goon

**Affiliations:** 1 Plastic and Reconstructive Surgery, Lister Hospital, Stevenage, GBR

**Keywords:** airless spray gun, concrete injection, reconstructive surgery, hand trauma, high-pressure injection

## Abstract

High-pressure injection injuries of the hand are uncommon but are associated with significant morbidity and require urgent surgical intervention. We describe a case of high-pressure injection of cement into the digit of a male patient while using an airless spray gun. We outline the initial assessment and surgical intervention, patient counselling regarding definitive management, and long-term outcomes of his injury. We also discuss mechanisms of high-pressure injection injuries, reconstructive options, and present a review of outcomes in patients sustaining similar injuries.

## Introduction

High-pressure injection injuries to the hand are infrequent but serious, often arising as a result of manual labour. These injuries predominantly occur in men around the age of 35 years [1], and typically involve the non-dominant hand index digit [2]. Injuries to the middle finger and palm are the second and third most common occurrences [1].

The severity of these injuries is often underestimated by patients and initial care providers. Severe symptoms are often not present immediately in the aftermath of the injury [3]. A high index of suspicion is required to recognise and treat these injuries appropriately. Urgent surgical debridement is indicated to remove injected material. This serves to arrest chemical injury to tissues, reducing further injury and preserving tissues. Despite early intervention, amputation rates as high as 58% have been reported [1]. In cases where amputation is not performed, just 43% are able to return to previous employment [4]. Given the potential long-term sequelae of high-pressure injection injuries, careful preoperative counselling regarding possible outcomes and prognosis is required when an effort is made to save the digit.

## Case presentation

The patient was a 31-year-old male, a right-hand-dominant construction worker who was referred to our plastic surgery unit from a local minor injuries unit. He complained of pain in his left ring finger, following an inadvertent injury with a spray gun containing Newton 103-S (a type of liquid cement). The patient had been wearing protective gloves at the time of injury. He had no past medical history, took no regular medications, and had no allergies.

**Figure 1:**
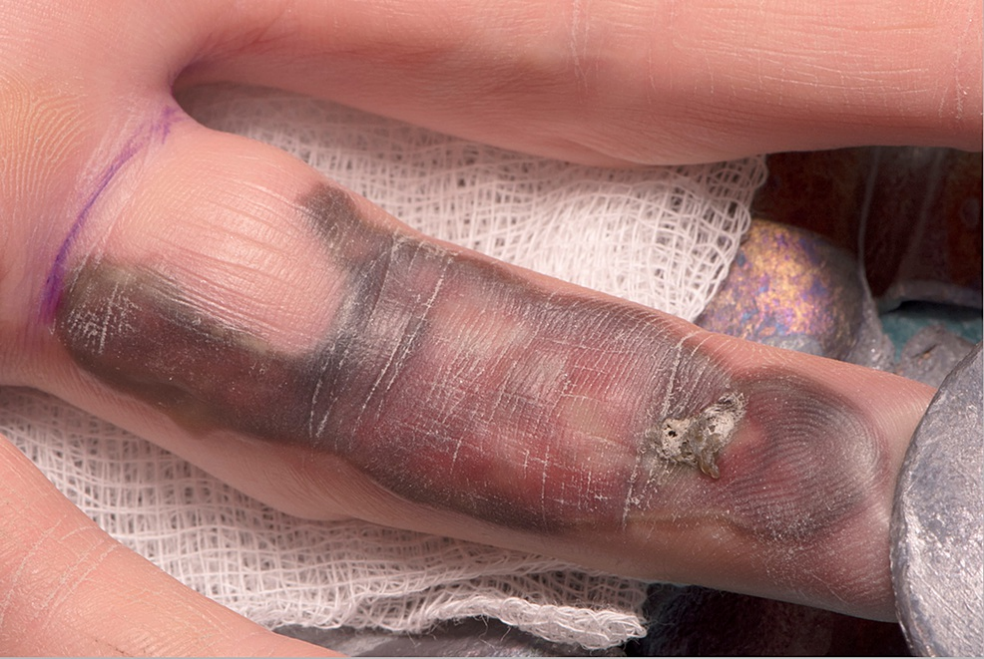
Volar surface of left ring finger at presentation

The patient had been given tetanus immunisation by the referring hospital. He had a plastic surgery review approximately three hours post-injury. On examination, his left ring finger was swollen, erythematous with necrotic skin on the volar aspect of the finger (Figure 1). There was no evidence of distal vascular compromise and the dorsal skin was well-perfused. He had a complete loss of sensation in the distribution of the ulnar digital nerve; however, the radial digital nerve was clinically intact. There was decreased range of movement at both the proximal interphalangeal joint (PIPJ) and distal interphalangeal joint (DIPJ). The palmar skin was not involved.

Blood results and observations were within normal ranges. No X-rays were taken at the time of presentation. Broad-spectrum IV antibiotics were commenced, and the patient was taken to theatre for urgent debridement and washout under general anaesthetic.

**Figure 2:**
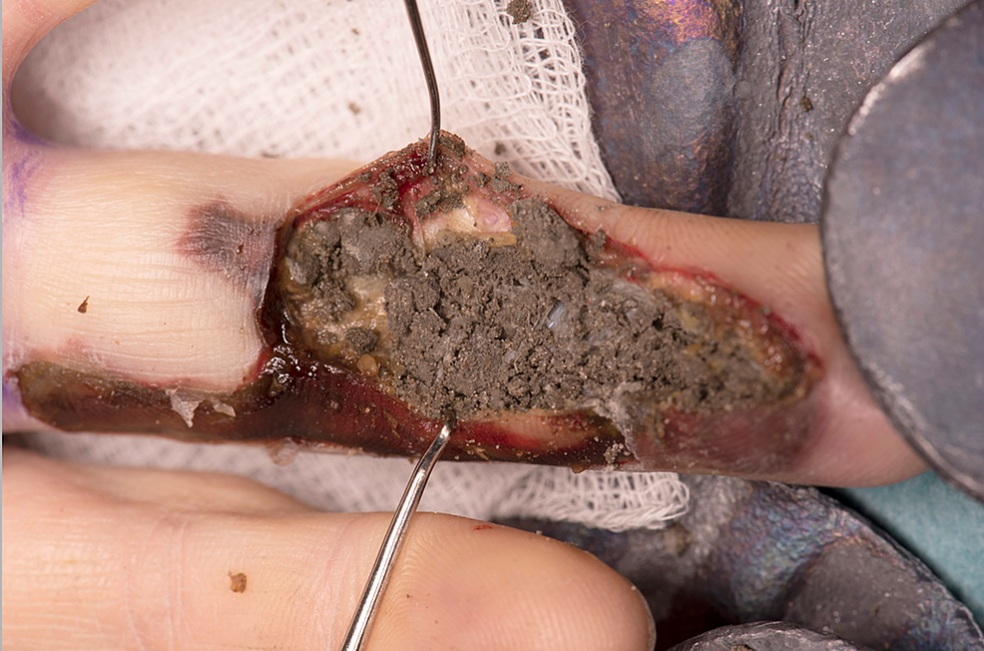
Hardened concrete within the subcutaneous tissues of the ring finger

The initial surgical approach consisted of Bruner incisions. Significantly hardened concrete was identified in the subcutaneous tissues (Figure 2). It was evident that skin and subcutaneous tissues were non-viable and were debrided, improving access. The pH of the wound was 8.5, in keeping with the alkaline substance injected; irrigation continued until pH returned to 7. The ulnar digital artery was thrombosed, but the radial digital artery was patent. The concrete had penetrated the flexor sheath, surrounding both flexor tendons. Subsequent flexor sheath washout from A1 to A5 confirmed that both tendons were in continuity but had undergone surface changes secondary to contact with the concrete.

**Figure 3:**
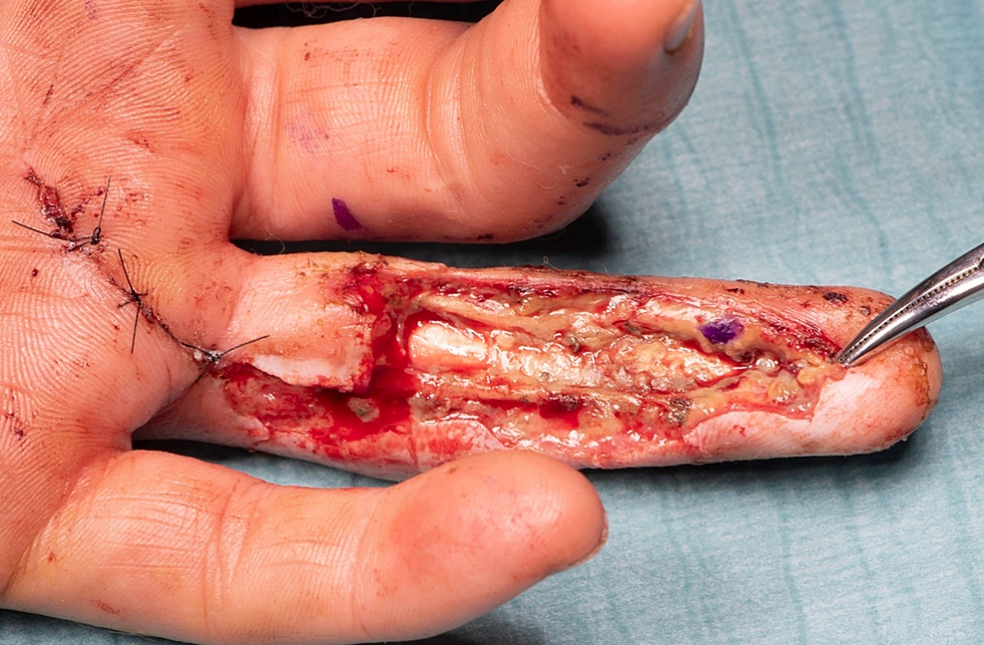
Ring finger post-debridement showing exposed flexor apparatus

A second look was performed at 48 hours; the distal digit remained viable with the flexor tendons appearing healthy. Tendon glide was impeded by friction within the flexor sheath. Access incision was made into the palm, which revealed further cement material. The passive range of movement was reassessed, which confirmed PIPJ and DIPJ to be in good condition. Further debridement of devitalised soft tissue resulted in a large defect of almost the full length of the volar aspect of the ring finger exposing the intact flexor apparatus pulleys and tendons (Figure 3).

The patient was counselled extensively regarding ongoing management, which principally consisted of either reconstruction of the volar soft tissue defect or potential amputation through the middle phalanx. He was counselled that the reconstructive option would result in a stiff and potentially insensate finger but could preserve length, while amputation might provide a faster recovery and better overall functional outcome. The patient opted for reconstruction and preservation of as much length of the digit as possible.

**Figure 4:**
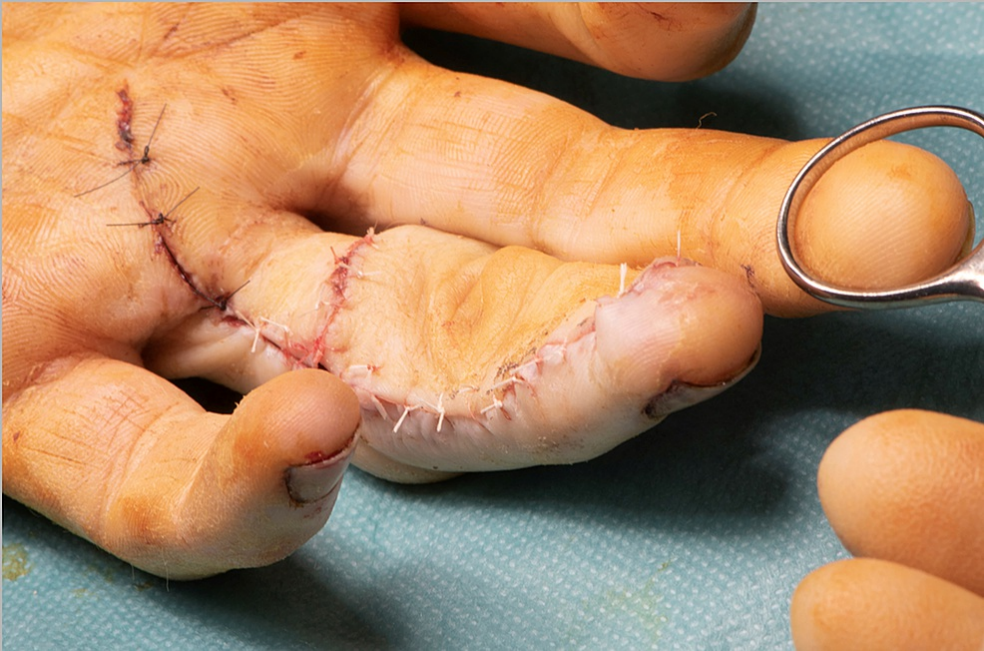
Cross-finger flap from the dorsum of the middle finger used to cover the volar defect of the ring finger

At one week from the initial injury, a cross-finger flap from the ipsilateral middle finger was performed, reconstructing the volar soft tissue defect (Figure 4). The patient was discharged with oral antibiotics.

After three weeks, the flap was divided and inset. However, the patient developed a wound infection five weeks after reconstruction, necessitating further washout and IV antibiotics.

**Figure 5:**
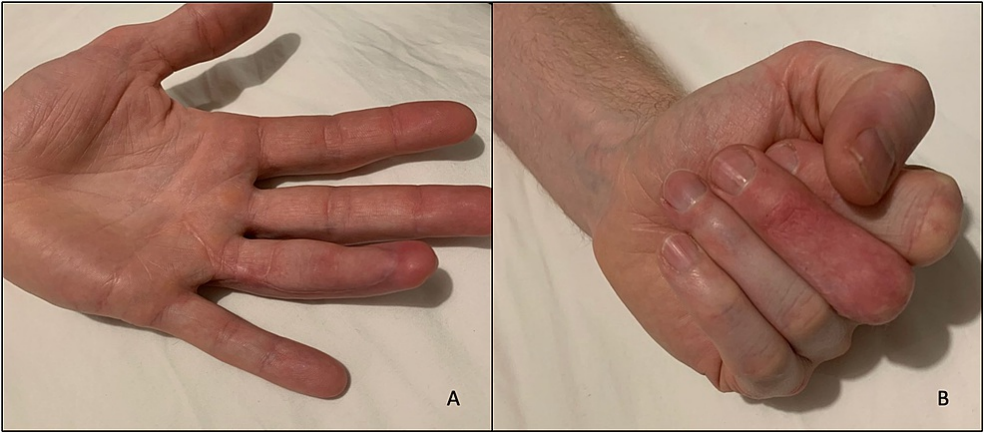
Postoperative photos at 18 months in full extension (A) and full flexion to make a fist (B)

At four months post-injury, the digit had an active range of movement of 20-84 degrees at the metacarpophalangeal joint (MCPJ); both PIPJ and DIPJ were in fixed 30 degrees of flexion. The protective sensation was maintained in the radial aspect of the digit, and the patient did not develop issues with pain subsequently. He was followed up via telephone consultation at 18 months postoperatively, and he reported that he had no functional limitations on daily activities, and was back to work. The skin overlying the volar defect of the ring finger and grafted area on the dorsum of the middle finger has healed well (Figures 5A, 5B).

## Discussion

Damage from high-pressure injection injury is thought to occur via four main mechanisms [5]: mechanical trauma from high-pressure; caustic injury from injected material; inflammatory reaction with oedema leading to further pressure injury; and infection as a complication from the initial injury.

Initially, the injected material spreads in its line of trajectory until reaching a tissue with sufficient strength to deflect it, such as bone, or annular ligaments. In cadaveric studies of injected wax, the injected material has been observed to take the path of least resistance, causing traumatic dissection that often spreads in subcutaneous tissues or along neurovascular bundles [6]. Injected material less commonly infiltrates the flexor sheath itself, due to the strength of the overlying annular ligaments; however, if the injected material encounters a weaker portion of the sheath, such as over the cruciate ligaments at a joint space, it can penetrate the sheath, causing distension and compression of the flexor tendon, as in this case.

Cement is an alkaline substance, which causes a reaction similar to a chemical burn [7], and prolonged exposure puts tissues at risk of severe necrosis. Injected cement is initially in a liquid state, making urgent washout more technically challenging as the potential for spread when washing out a fluid material needs to be balanced against potential neurovascular compromise and ongoing chemical irritation if it is left to harden [8].

The inflammatory reaction to injected material and injury causes increasing oedema, thereby compromising the blood flow to the injured digit. The time between injury and definitive treatment in the form of surgical debridement has been found to be the most important prognostic factor [1], as early surgical debridement interrupts the ongoing caustic injury and decompression of the digit counteracts the compartment syndrome effect of increasing swelling within a non-distensible digit.

Infection complicates some cases of high-pressure injection injuries, with areas of tissue necrosis acting as a nidus for polymicrobial infection. For this reason, broad-spectrum antibiotics are often used. As in this case, the presentation of infection can be delayed, as often the injected material itself is inert, and hence it takes time for tissue necrosis and ischaemia to allow infection to take hold [1].

In our review of the literature, we found just five cases describing the injection of cement or cement-containing substances into the hand. A published case series of three injuries resulting from a faulty high-pressure cement hose injecting cement at around 3000 psi describes poor outcomes [9]. In the first case, inadvertent injection entered into the pulp of the thumb, and symptoms subsided within 48 hours. It was managed conservatively, but the patient developed a discharging wound 28 days later. The second case involved a delayed presentation by the patient, approximately five weeks post-injury, again with infection. This patient underwent amputation of his finger at the PIPJ. In the third case, the flexor sheath of the index finger was contaminated with cement, and the patient underwent urgent surgical debridement. This was complicated by a postoperative infection that necessitated amputation at the MCPJ five weeks later.

More recently, in 2002, Barr et al. [8] described a case of injection into the first dorsal web space, which was treated with an urgent two-step debridement and washout; while the patient avoided amputation, it resulted in the incomplete recovery of function. The authors noted that undertaking the debridement of cement when it was still in a liquid state may have made the washout more difficult. Our case was delayed by 12 hours due to theatre unavailability, which may have given time for the cement to harden sufficiently for a less difficult washout. The risk of delaying washout in these cases is that prolonged exposure of the tissues to the alkaline fluid allows for a further caustic injury, which can lead to severe tissue necrosis, and thus may increase the likelihood of amputation.

One study has reported a late presentation of high-pressure cement injection over the hypothenar eminence, where the recognition of the severity of the injury had been delayed by the local emergency department, and cement particles were found to have migrated along the fascial planes to the mid-forearm, from the initial injection site [10]. This necessitated the resection of multiple flexor tendons and the ulnar artery and nerve with appropriate reconstructive grafting. The anatomy of this part of the hand and forearm allows for this proximal spread, in that the sheath is continuous. Injections over the palm and index to ring fingers do not usually have the same propensity for propagation.

Thumb and hand injuries result in amputation less frequently compared to those in fingers [1], which may reflect a greater potential functional benefit if conservative management is successful. Early amputation has previously been encouraged in severely injured digits, as it can be associated with a shorter period of morbidity and quicker return to work [6].

To the authors' knowledge, this is the first case to describe a scenario where the injection of high-pressure cement into a finger has undergone urgent surgical debridement, and amputation has been avoided. Patients who sustain such injuries are often manual workers, and hence a good functional outcome with respect to the finger is key. In patients for whom preservation of the full length of the finger is desired, careful preoperative counselling about potential postoperative sequelae such as neuropathic pain, cold intolerance, and significant stiffness is essential [11].

## Conclusions

Emergency and primary care providers should be aware that high-pressure injection injuries can present with mild symptoms at first; hence, a high index of suspicion is needed in order to recognise and treat them expediently. Workup for suspected high-pressure injection injuries should include X-rays, tetanus prophylaxis, broad-spectrum antibiotics, and early referral to Hand Surgery.

Most high-pressure injection injuries will benefit from early surgical debridement, to reduce the risk of ongoing tissue damage due to the ischaemic-inflammatory reaction caused by injected foreign bodies. However, despite early debridement, many patients who have had high-pressure injection injuries may have significant postoperative morbidity. Careful preoperative counselling is necessary in cases of severely injured digits, as functional outcomes are variable, and a decision as to the conservation of full length of the digit with reduced function versus early amputation must be made on a case-by-case basis.
